# Risk factors for lateral cervical lymph node metastasis in papillary thyroid carcinoma and to develop and validate a nomogram model

**DOI:** 10.3389/fendo.2025.1730943

**Published:** 2025-12-15

**Authors:** Lei An, Anran Du, Jiayi Wang, Xiaopei Li, Ning Zhao, Zhicheng Ge, Guoqian Ding

**Affiliations:** 1 Department of General Surgery, Beijing Friendship Hospital, Capital Medical University, Beijing, China; 2 Department of General Surgery Center, Beijing Friendship Hospital, Capital Medical University, Beijing, China

**Keywords:** papillary thyroid carcinoma, lateral lymph node metastasis, risk factors, predictive model, nomogram

## Abstract

**Objective:**

To identify risk factors for lateral lymph node metastasis (LLNM) in papillary thyroid carcinoma (PTC) and to establish clinical prediction models.

**Methods:**

We retrospectively collected clinical data from 249 patients with PTC and suspected LLNM, 222 patients met the inclusion criteria. Based on postoperative pathology of the lateral compartment, 145 patients without metastasis were classified as the non-metastasis group, 77 patients with metastasis were classified as the metastasis group. All included patients were randomly assigned to training set and validation set. Univariate and multivariate logistic regression analyses were performed to screen predictors of LLNM and construct nomogram models for preoperative and postoperative prediction. Model performance was evaluated using the Hosmer-Lemeshow goodness-of-fit test, calibration curves with bootstrap resampling, receiver operating characteristic (ROC) curves and the area under the curve (AUC), as well as decision curve analysis (DCA).

**Results:**

In preoperative analyses, age, maximum tumor diameter ≥1 cm on ultrasound, hyperechoic area in the lateral cervical lymph node, and lateral cervical lymph nodes perinodal vascularity were independent predictors of LLNM. In postoperative analyses, age, multifocality, pathological maximum tumor diameter ≥1 cm, and concomitant central lymph node metastasis were independent predictors. The AUCs for the preoperative model were 0.805 (training set) and 0.719 (validation set), and for the postoperative model were 0.885 (training set) and 0.762 (validation set). After 1,000 bootstrap resamples, the mean absolute errors (MAE) of the calibration curves were 0.047 and 0.066 for the preoperative model (training set and validation set), and 0.021 and 0.046 for the postoperative model.

**Conclusion:**

DCA showed a higher net clinical benefit of both models than the treat-all or treat-none strategies, indicating good predictive accuracy and clinical utility.

## 1 Introduction

Thyroid carcinoma (TC) is the most common malignant neoplasm of the endocrine system. Papillary thyroid carcinoma (PTC) is the predominant histologic subtype, accounting for approximately 70%–90% of cases, and is the only histologic variant that has shown a sustained increase in incidence in recent years (1). Although PTC is generally considered a low-grade malignancy with a favorable prognosis, 30%–80% of patients present with cervical lymph node metastasis (LNM) (2). LNM can be divided into central lymph node metastasis (CLNM) and lateral lymph node metastasis (LLNM). LNM has been demonstrated to be an important adverse prognostic factor in PTC, being closely associated with distant metastasis, recurrence (3), and unfavorable outcomes, LLNM has been particularly linked to local recurrence and survival (4).

Surgery remains the mainstay of treatment for PTC, especially in patients with nodal disease. Accurate preoperative assessment of LNM helps define the extent and strategy of surgery and is crucial for optimizing the operative plan. Therapeutic lateral neck dissection (LND) can remove metastatic lymph nodes to control locoregional disease progression and reduce recurrence risk, but it increases the risks of complications such as postoperative bleeding, chylous leakage, nerve injury, and hypoparathyroidism (5). Currently, guidelines recommend therapeutic LND for LLNM only when there is a high preoperative suspicion or cytologic/histopathologic confirmation (6, 7).

In this study, we aimed to determine independent clinical risk factors for LLNM in PTC and to develop both preoperative and postoperative prediction models to assist surgeons with preoperative planning and postoperative follow-up.

## 2 Materials and methods

### 2.1 Study population

We retrospectively collected clinical data from 249 patients with PTC and suspected LLNM who were treated at Beijing Friendship Hospital between January 2020 and July 2023.

### 2.2 Inclusion criteria

1. Thyroid function testing and thyroid ultrasound performed within 1 month before surgery,
2. First thyroid operation,
3. Postoperative paraffin pathology confirming PTC,
4. Complete clinical data available.

### 2.3 Exclusion criteria

1. Postoperative paraffin pathology indicating other histologic types of thyroid malignancy,
2. Concomitant malignancies of other organ systems,
3. History of prior neck surgery,
4. Incomplete clinical data.

### 2.4 Data collection

The following variables were collected: sex, age, and coexisting Hashimoto’s thyroiditis (HT), preoperative color Doppler ultrasound of the thyroid and cervical lymph nodes within 1 month before surgery, including: laterality of the thyroid nodule (unilateral/bilateral), intraglandular location, aspect ratio, maximum diameter on ultrasound, calcification, capsular relation, shape regular/slightly regular, intratumoral blood flow, clarity of the lymphatic hilum in suspicious lymph nodes, hyperechoic area in the lateral cervical lymph node, and lateral cervical lymph nodes perinodal vascularity, postoperative paraffin pathology: BRAF V600E mutation, multifocality, pathological maximum tumor diameter, perineural and vascular invasion, lymphatic invasion, and presence of CLNM.

### 2.5 Study design and statistical methods

Based on the inclusion and exclusion criteria, 222 patients were finally included. Patients were categorized into the LLNM group (n=77) and the non-LLNM group (n=145) according to pathological confirmation of lateral compartment metastasis. All 222 patients were randomly assigned at a 7:3 ratio to a training set (n=155) and a validation set (n=67), and baseline comparability between sets was assessed. Variables were subjected to univariate logistic regression, and those with P<0.05 were entered into multivariate logistic regression to identify independent predictors of LLNM. A nomogram was constructed in R based on the independent factors. Discrimination was assessed by ROC analysis and AUC. Calibration was assessed using the Hosmer-Lemeshow goodness-of-fit test and calibration curves with 1,000-time bootstrap resampling. Clinical utility was evaluated using decision curve analysis (DCA).

**Figure 1:**
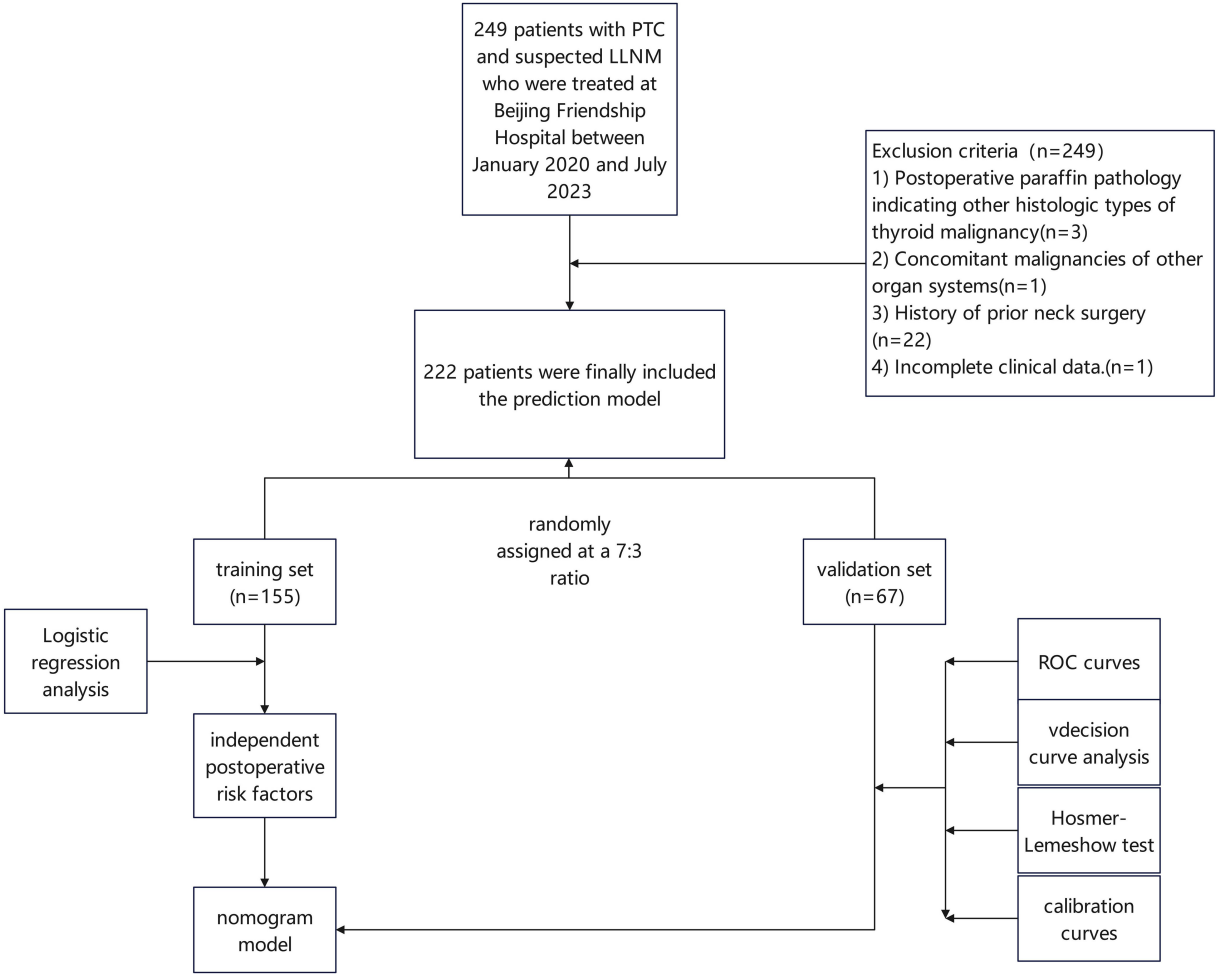
Flow chart of case screening.

Statistical analysis was performed with IBM SPSS Statistics 27.0 and R 4.4.1. Categorical variables are presented as frequency (percentage) and compared using the chi-square (χ²) test. Univariate logistic regression was conducted for variables stratified by LLNM status, variables with P<0.05 entered multivariate logistic regression to identify independent predictors (P<0.05). LASSO regression using the ‘glmnet’ package was applied to screen common risk factors. The clinical models and nomograms were built using the ‘rms’ package, ROC curves and AUC were calculated with ‘pROC’, calibration curves were drawn with ‘rms’, and DCA was performed with the ‘rmda’ package (**Figure 1**).

## 3 Results

According to the eligibility criteria, 222 patients were finally included and divided into the LLNM group (n=77) and the non-LLNM group (n=145). In the overall set, males and females accounted for 33.10% and 66.90%, respectively; 29.66% of patients were aged ≥45 years; HT was present in 26.90%; bilateral lesions in 38.62%; ultrasound findings included nodule calcification in 88.28%, capsular involvement in 62.76%, shape slightly regular in 76.55%, boundary slightly clear in 75.17%, aspect ratio ≥1 in 37.93%, and blood flow in 66.21%; the lesion was located in the upper pole in 24.83%; maximum tumor diameter on ultrasound ≥1 cm in 73.79%; suspicious lateral cervical lymph nodes showed unclear lymphatic hilum in 64.14%, hyperechoic areas in 61.38%, and perinodal vascularity in 53.10%. Postoperative pathology revealed BRAF V600E mutation in 79.31%, multifocality in 50.34%, pathological maximum diameter ≥1 cm in 71.72%, perineural/vascular invasion in 27.59%, lymphatic invasion in 29.66%, and concomitant CLNM in 91.03% (**Table 1**).

**Table 1:** Comparison of the clinicopathological characteristics between LLNM and NLLNM group of the PTC patients.

Variables	Group	LLNM (n=145)	Non-LNMM (n=77)	χ2	P
Gender	Male	48 (33.10)	18 (23.38)	2.278	0.131
	Female	97 (66.90)	59 (76.62)		
Age (years)	<45	102 (70.34)	35 (45.45)	13.187	<0.001
	≥45	43 (29.66)	42 (54.55)		
Combined Hashimoto’s thyroiditis	Yes	39 (26.90)	30 (38.96)	3.417	0.065
	No	106 (73.10)	47 (61.04)		
Affected side	Unilateral	89 (61.38)	50 (64.94)	0.272	0.602
	Bilateral	56 (38.62)	27 (35.06)		
Ultrasound features					
Calcification	Yes	128 (88.28)	58 (75.32)	6.209	0.013
	No	17 (11.72)	19 (24.68)		
Capsular relation	Yes	91 (62.76)	49 (63.64)	0.017	0.897
	No	54 (37.24)	28 (36.36)		
Shape	Regular	34 (23.45)	17 (22.08)	0.053	0.817
	Slightly regular	111 (76.55)	60 (77.92)		
Boundary	Clear	36 (24.83)	16 (20.78)	0.460	0.498
	Slightly clear	109 (75.17)	61 (79.22)		
Aspect ratio	<1	90 (62.07)	32 (41.56)	8.547	0.003
	≥1	55 (37.93)	45 (58.44)		
Blood flow	Yes	96 (66.21)	45 (58.44)	1.309	0.253
	No	49 (33.79)	32 (41.56)		
Location	Upper	36 (24.83)	8 (10.39)	12.489	0.014
	Middle	57 (39.31)	29 (37.66)		
	Lower	19 (24.68)	15 (10.34)		
	Isthmus	2 (2.60)	2 (1.38)		
	Multifocality	19 (24.68)	35 (24.14)		
Maximum diameter under Ultrasound (cm)	<1	38 (26.21)	42 (54.55)	17.522	<0.001
	≥1	107 (73.79)	35 (45.45)		
Lymphatic hilum	Clear	52 (35.86)	20 (25.97)	2.244	0.134
	Slightly clear	93 (64.14)	57 (74.03)		
Hyperechoic area in the lymph node	Yes	89 (61.38)	25 (32.47)	16.828	<0.001
	No	56 (38.62)	52 (67.53)		
Perinodal vascularity	Yes	77 (53.10)	23 (29.87)	10.967	0.001
	No	68 (46.90)	54 (70.13)		
Pathology					
BRAF V600E mutation	Yes	115 (79.31)	64 (83.12)	0.467	0.495
	No	30 (20.69)	13 (16.88)		
Multifocality	Yes	73 (50.34)	27 (35.06)	4.743	0.029
	No	72 (49.66)	50 (64.94)		
pathological Maximum diameter (cm)	<1	41 (28.28)	50 (64.94)	27.942	<0.001
	≥1	104 (71.72)	27 (35.06)		
Perineural/Vascular invasion	Yes	40 (27.59)	13 (16.88)	3.170	0.075
	No	105 (72.41)	64 (83.12)		
Lymphatic invasion	Yes	43 (29.66)	11 (14.29)	6.454	0.011
	No	102 (70.34)	66 (85.71)		
Central cervical lymph node metastasis	Yes	132 (91.03)	32 (41.56)	63.787	<0.001
	No	13 (8.97)	45 (58.44)		

At a 7:3 ratio, the entire set was randomly divided into a training set (n=155) and a validation set (n=67). The rates of LLNM were 65.81% and 64.18% in the training and validation sets, respectively, with no statistically significant differences (P = 0.815). There were no significant differences between sets in general characteristics, preoperative laboratory tests, or postoperative pathology (all P>0.05), indicating baseline comparability (**Table 2**).

**Table 2:** Comparison of baseline data between training and validation groups.

Variables		Training group	Validation group	χ2	P
LLNM	Yes	102 (65.81	43 (64.18)	0.055	0.815
	No	53 (34.19	24 (35.82)		
Gender	Male	45 (29.03)	21 (31.34)	0.120	0.729
	Female	110 (70.97)	46 (68.66)		
Age (years)	<45	97 (62.58)	40 (59.70)	0.164	0.685
	≥45	58 (37.42)	27 (40.30)		
Combined Hashimoto’s thyroiditis	Yes	54 (34.84)	15 (22.39)	3.385	0.066
	No	101 (65.16)	52 (77.61)		
Affected side	Unilateral	93 (60.00)	46 (68.66)	1.498	0.221
	Bilateral	62 (40.00)	21 (31.34)		
Ultrasound features					
Calcification	Yes	132 (85.16)	54 (80.60)	0.717	0.397
	No	23 (14.84)	13 (19.40)		
Capsular relation	Yes	94 (60.65)	46 (68.66)	1.289	0.256
	No	61 (39.35)	21 (31.34)		
Shape	Regular	36 (23.23)	20 (29.85)	2.565	0.109
	Slightly regular	119 (76.77)	47 (70.15)		
Boundary	Clear	36 (23.23)	16 (23.88)	0.011	0.916
	Slightly clear	119 (76.77)	51 (76.12)		
Aspect ratio	<1	86 (55.48)	36 (53.73)	0.058	0.810
	≥1	69 (44.52)	31 (46.27)		
Blood flow	Yes	98 (63.23)	43 (64.18)	0.018	0.892
	No	57 (36.77)	24 (35.82)		
Location	Upper	34 (21.94)	10 (14.93)	8.991	0.061
	Middle	64 (41.29)	22 (32.84)		
	Lower	18 (11.61)	16 (23.88)		
	Isthmus	4 (2.58)	0 (0.00)		
	Multifocality	35 (22.58)	19 (28.36)		
Maximum diameter under ultrasound (cm)	<1	55 (35.48)	25 (37.31)	0.068	0.794
	≥1	100 (64.52)	42 (62.69)		
Lymphatic hilum	Clear	53 (34.19)	19 (28.36)	0.727	0.394
	Slightly clear	102 (65.81)	48 (71.64)		
Hyperechoic area in the lymph node	Yes	83 (53.55)	31 (46.27)	0.992	0.319
	No	72 (46.45)	36 (53.73)		
Perinodal vascularity	Yes	68 (43.87)	32 (47.76)	0.286	0.593
	No	87 (56.13)	35 (52.24)		
Pathology					
BRAF V600E mutation	Yes	125 (80.65)	54 (80.60)	0.000	0.993
	No	30 (19.35)	13 (19.40)		
Multifocality	Yes	73 (47.10)	27 (40.30)	0.873	0.350
	No	82 (52.90)	40 (59.70)		
pathological Maximum diameter (cm)	<1	62 (40.00)	29 (43.28)	0.209	0.648
	≥1	93 (60.00)	38 (56.72)		
Perineural/Vascular invasion	Yes	36 (23.23)	17 (25.37)	0.119	0.730
	No	119 (76.77)	50 (74.63)		
Lymphatic invasion	Yes	39 (25.16)	15 (22.39)	0.195	0.658
	No	116 (74.84)	52 (77.61)		
Central cervical lymph node metastasis	Yes	114 (73.55)	50 (74.63)	0.028	0.867
	No	41 (26.45)	17 (25.37)		

Logistic regression analysis: Univariate analysis showed that age, tumor aspect ratio ≥1 on ultrasound, maximum tumor diameter on ultrasound, clarity of lateral cervical lymphatic hilum, hyperechoic area in the lateral cervical lymph node, and lateral cervical lymph nodes perinodal vascularity; as well as multifocality, pathological maximum diameter, lymphatic invasion, and concomitant CLNM were associated with LLNM (**Table 3**). Multivariate logistic regression identified age, maximum tumor diameter on ultrasound, hyperechoic area in the lateral cervical lymph node, and lateral cervical lymph nodes perinodal vascularity as independent preoperative risk factors (**Table 4**); and age, multifocality, pathological maximum diameter, and concomitant CLNM as independent postoperative risk factors (**Table 5**).

**Table 3:** Univariate analysis logistic regression analysis for clinical factors associated with LLNM.

Factors	β	Standard error	z	Wald χ2	p	OR	95% CI
Gender	0.341	0.384	0.888	0.789	0.374	1.407	0.663 ~ 2.986
Age(years)	-0.989	0.351	-2.820	7.953	0.005	0.372	0.187 ~ 0.740
Combined Hashimoto’s thyroiditis	-0.563	0.351	-1.604	2.574	0.109	0.570	0.286 ~ 1.133
Affected side	0.514	0.355	1.446	2.090	0.148	1.672	0.833 ~ 3.356
Calcification	0.465	0.460	1.011	1.023	0.312	1.592	0.647 ~ 3.921
Capsular relation	0.137	0.345	0.396	0.157	0.692	1.146	0.583 ~ 2.256
Shape	-0.109	0.428	-0.254	0.064	0.800	0.897	0.388 ~ 2.076
Boundary	-0.386	0.418	-0.923	0.852	0.356	0.680	0.300 ~ 1.543
Aspect ratio	-0.745	0.344	-2.167	4.694	0.030	0.475	0.242 ~ 0.931
Blood flow	0.063	0.350	0.179	0.032	0.858	1.065	0.536 ~ 2.115
Location	-0.066	0.117	-0.567	0.321	0.571	0.936	0.745 ~ 1.176
Maximum diameter under ultrasound (cm)	1.391	0.360	3.860	14.899	<0.001	4.017	1.983 ~ 8.140
Lymphatic hilum	-0.831	0.385	-2.157	4.654	0.031	0.435	0.205 ~ 0.927
Hyperechoic area in the lymph node	1.488	0.365	4.079	16.637	<0.001	4.427	2.166 ~ 9.048
Perinodal vascularity	0.750	0.355	2.115	4.474	0.034	2.118	1.057 ~ 4.244
BRAF V600E mutation	-0.048	0.431	-0.111	0.012	0.912	0.953	0.410 ~ 2.218
Multifocality	0.947	0.355	2.666	7.107	0.008	2.578	1.285 ~ 5.172
pathological Maximum diameter (cm)	1.823	0.372	4.903	24.042	<0.001	6.190	2.987 ~ 12.828
Perineural/Vascular invasion	0.755	0.443	1.704	2.904	0.088	2.128	0.893 ~ 5.074
Lymphatic invasion	0.899	0.440	2.042	4.171	0.041	2.456	1.037 ~ 5.817
Central cervical lymph node metastasis	2.757	0.448	6.153	37.855	<0.001	15.746	6.543 ~ 37.891

**Table 4:** Multivariate logistic regression analysis for clinical factors associated with LLNM.

Factors	β	Standard error	z	Wald χ2	p	OR	95% CI
Age(years)	-1.165	0.423	-2.757	7.600	0.006	0.312	0.136 ~ 0.714
Aspect ratio	-0.766	0.418	-1.833	3.361	0.067	0.465	0.205 ~ 1.054
Maximum diameter under ultrasound (cm)	1.571	0.424	3.704	13.719	<0.001	4.812	2.095 ~ 11.051
Lymphatic hilum	-0.637	0.449	-1.419	2.013	0.156	0.529	0.219 ~ 1.275
Perinodal vascularity	1.471	0.415	3.540	12.532	<0.001	4.352	1.928 ~ 9.825
Hyperechoic area in the lymph node	0.935	0.432	2.167	4.697	0.030	2.548	1.094 ~ 5.938

**Table 5:** Multivariate logistic regression analysis for clinicopathologic factors associated with LLNM.

Factors	Regression coefficient	Standard error	z	Wald χ2	p	OR	95% CI
Age(years)	-1.051	0.492	-2.134	4.554	0.033	0.350	0.133 ~ 0.918
Multifocality	1.309	0.497	2.635	6.942	0.008	3.703	1.398 ~ 9.808
pathological Maximum diameter (cm)	1.737	0.462	3.763	14.163	<0.001	5.680	2.299 ~ 14.036
Lymphatic invasion	0.682	0.530	1.286	1.655	0.198	1.978	0.700 ~ 5.592
Central cervical lymph node metastasis	2.500	0.516	4.840	23.429	<0.001	12.177	4.426 ~ 33.504

**Figure 2:**
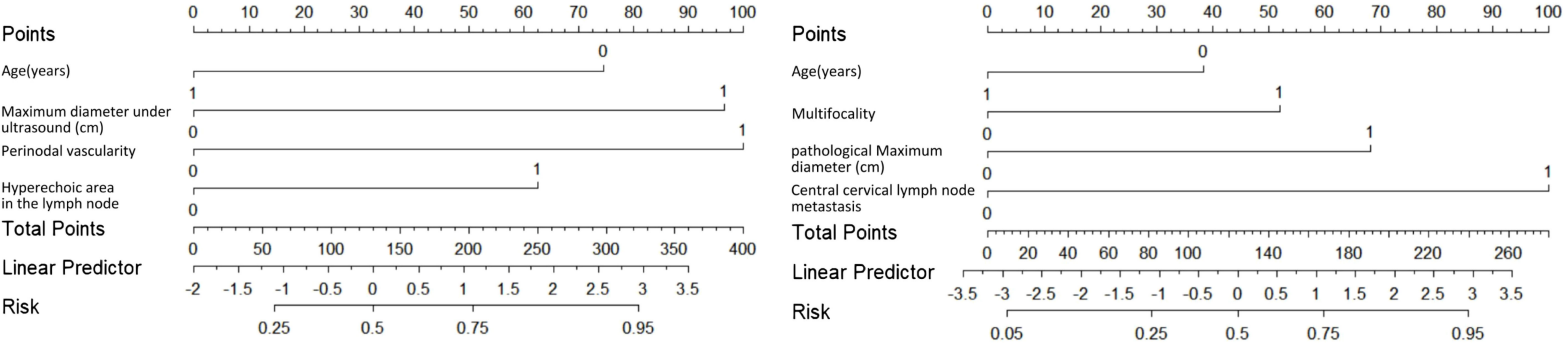
Nomograms for predicting the probability of LLNM in patients with PTC. **(A)** Preoperative model based on preoperative clinical factors; **(B)** Postoperative model based on clinicopathological factors.

Based on the multivariate analyses, two nomogram models were developed: Model A (preoperative), incorporating four preoperative predictors—age, maximum tumor diameter on ultrasound, presence of hyperechoic areas in suspicious lateral cervical lymph nodes, and lateral cervical lymph nodes perinodal vascularity; and Model B (postoperative), incorporating four postoperative predictors—age, multifocality, pathological maximum diameter, and concomitant CLNM (**Figure 2**).

**Figure 3:**
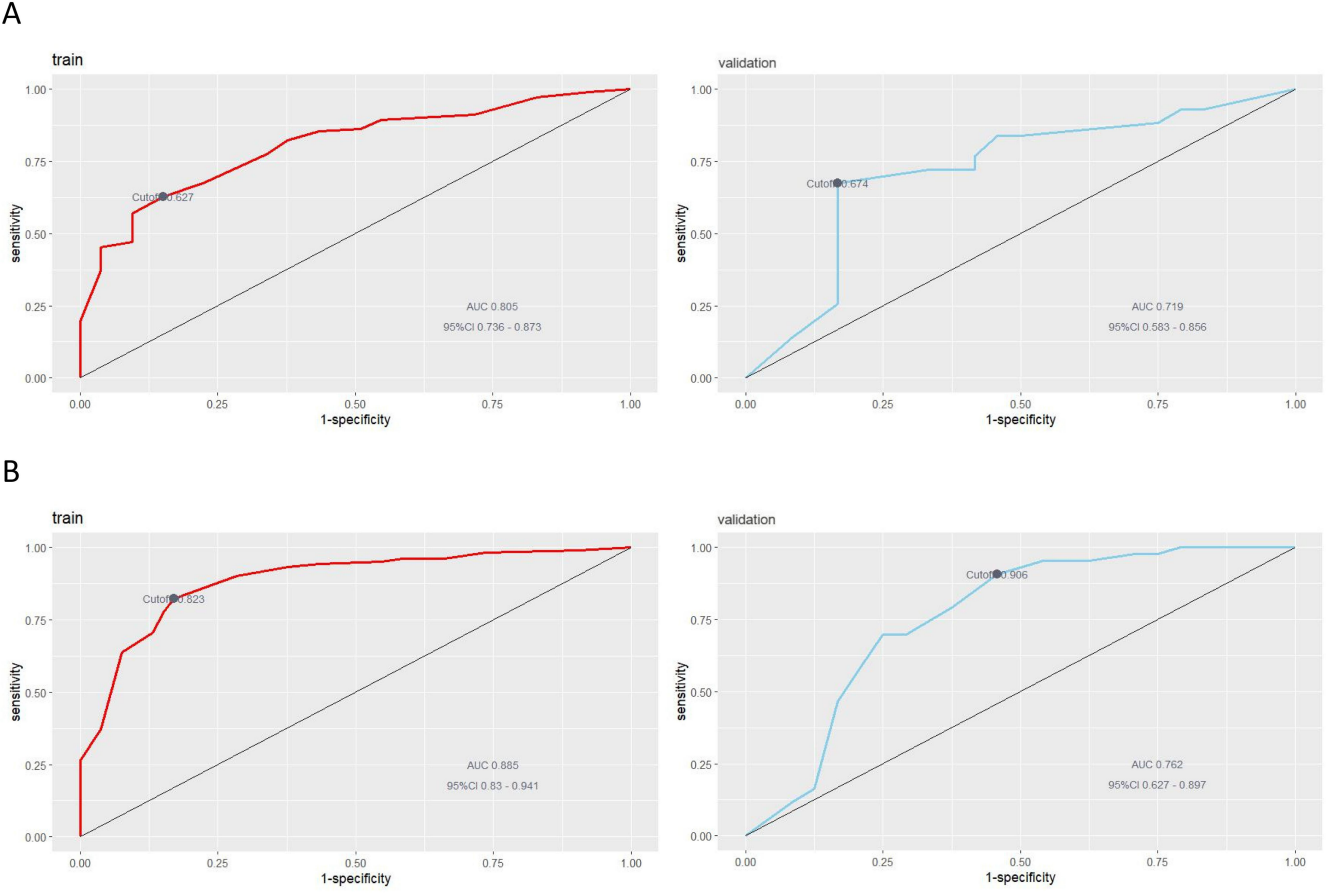
ROC curve analysis to predict LLNM in PTC patients. **(A)** ROC curve analysis based on preoperative clinical factors; **(B)** ROC curve analysis based on clinicopathological factors.

Model discrimination: In the preoperative model, the AUCs were 0.805 (95% CI, 0.736–0.873) for the training set and 0.719 (95% CI, 0.583–0.856) for the validation set; optimal cutoff values were 0.627 and 0.674, respectively. In the postoperative model, the AUCs were 0.885 (95% CI, 0.830–0.941) for the training set and 0.762 (95% CI, 0.627–0.897) for the validation set; optimal cutoff values were 0.823 and 0.906, respectively. These findings indicate good discrimination of both models for stratifying LLNM risk (**Figure 3**).

**Figure 4:**
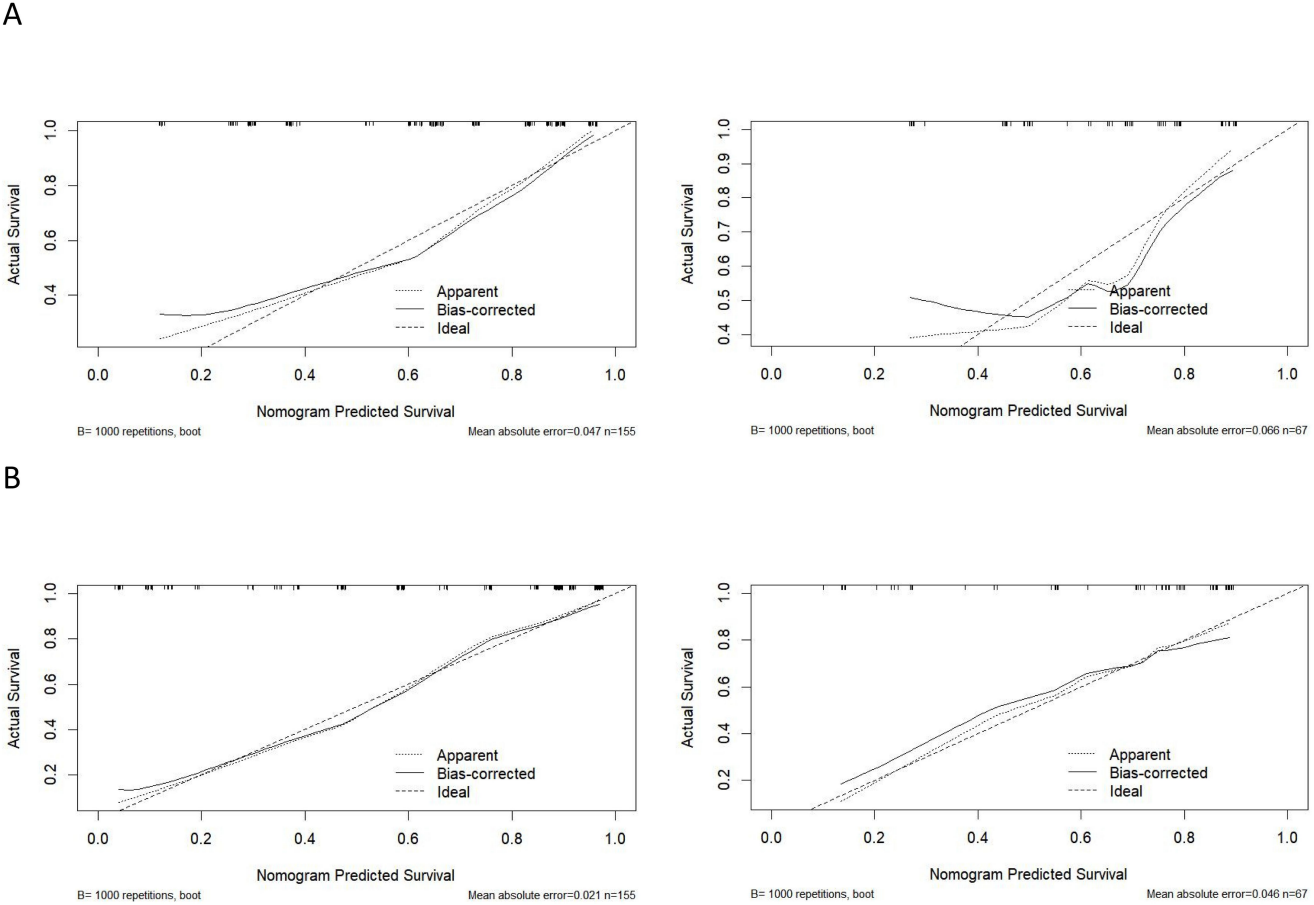
Calibration curve of the nomogram. **(A)** Calibration curve of the nomogram based on preoperative clinical factors; **(B)** Calibration curve of the nomogram based on clinicopathological factors.

Model calibration: The Hosmer-Lemeshow goodness-of-fit tests showed X²=6.729 (P = 0.457) and X²=7.566 (P = 0.372) for the preoperative model in the training and validation sets, respectively; and X²=3.743 (P = 0.711) and X²=2.476 (P = 0.780) for the postoperative model in the training and validation sets, respectively. All P values exceeded 0.05, indicating good calibration of both models. Using 1,000-time bootstrap resampling, the MAE values were 0.047 and 0.066 for the preoperative model (training and validation), and 0.021 and 0.046 for the postoperative model. In calibration plots, the x-axis denotes the predicted probability of LLNM and the y−axis denotes the observed probability. The diagonal dashed line represents the ideal model, and the solid line represents the performance of the present model. The closer the solid line is to the diagonal, the better the predictive performance. For both models and in both sets, the calibration curves closely approximated the diagonal, demonstrating high accuracy for predicting LLNM risk (**Figure 4**).

**Figure 5:**
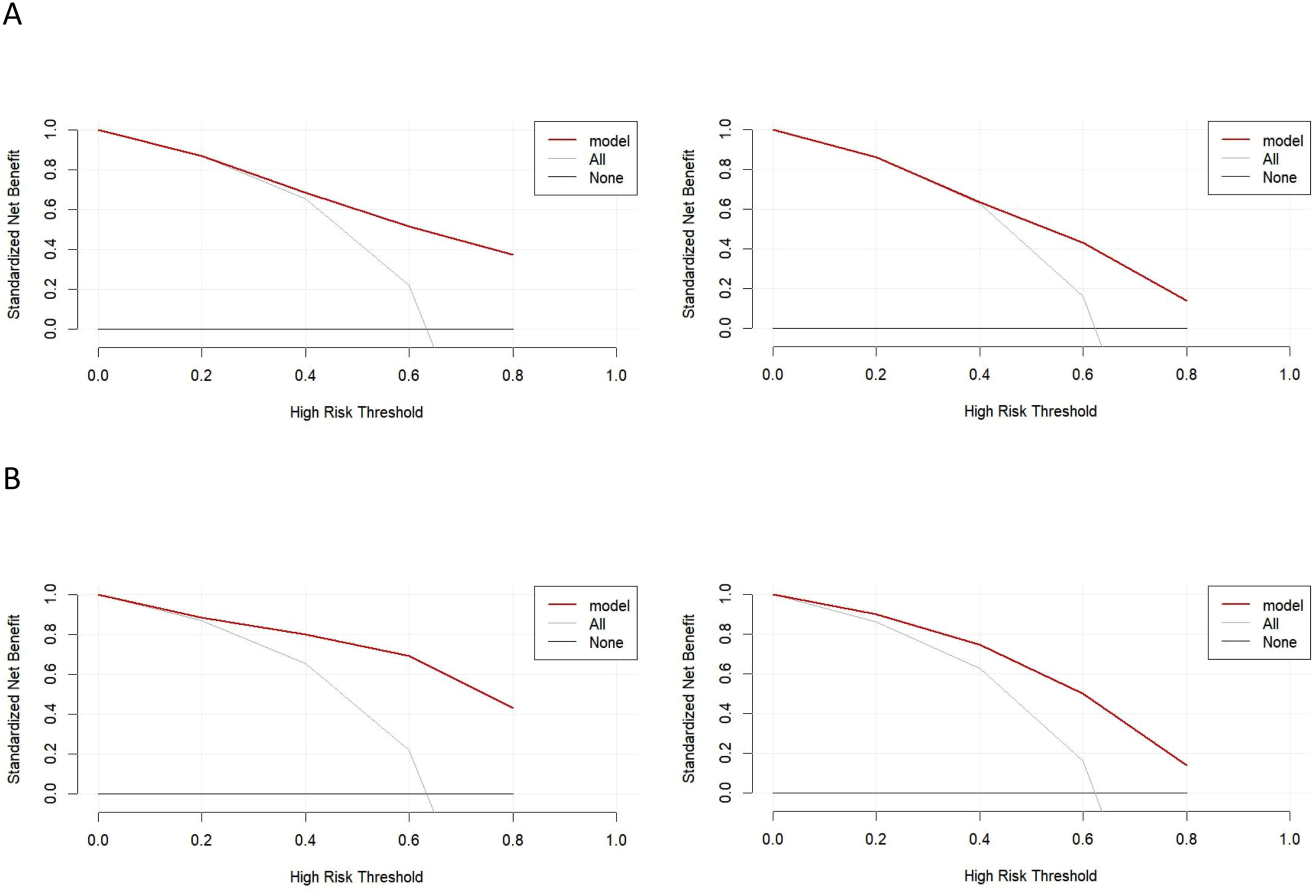
Decision curve analysis for the nomogram. **(A)** Decision curve analysis for the nomogram based on preoperative clinical factors; **(B)** Decision curve analysis for the nomogram based on clinicopathological factors.

Clinical utility: DCA was undertaken to evaluate the clinical usefulness of the models by considering the range of threshold probabilities at which interventions would be undertaken. The gray line denotes the strategy of treating all patients, the black line denotes treating none, and the red line denotes using the prediction model to guide management. In this study, the decision curves showed broad threshold ranges with positive net benefit, supporting the clinical applicability of the models and their value for decision-making (**Figure 5**).

## 4 Discussion

In PTC, lymphatic spread is the predominant metastatic pathway. The lateral cervical compartment is the second most frequent site of nodal involvement after the central compartment (2). Multiple studies have shown that LLNM is a major risk factor for local recurrence and poor prognosis in PTC (8–10). Preoperative assessment of LLNM risk enables surgeons to tailor the extent of surgery and the scope of lymph node dissection, improve oncologic outcomes, and reduce unnecessary surgical morbidity (11). Therefore, accurate preoperative identification of LLNM is of great clinical importance. Although CT is more sensitive than ultrasound for detecting LLNM, it is less specific (12, 13); fine-needle aspiration cytology also has a false-negative rate of approximately 30% (14), and occult LLNM may go undetected preoperatively (15). Thus, an objective and accurate tool to assess the likelihood of LLNM is needed.

The nomogram was adopted as it provides an intuitive and clinically practical tool for risk prediction. Its key advantage lies in visually representing the weight of each predictive factor through segment length, allowing clinicians to easily estimate an individual’s risk probability through simple summation without complex calculations. This model was specifically developed based on the significant predictors identified from our own dataset through multivariate logistic regression analysis, ensuring its direct relevance to our research context and clinical application.

In this retrospective study, we found that younger age, larger maximum tumor diameter on ultrasound, hyperechoic areas and vascularity in suspicious lateral cervical lymph nodes, as well as postoperative multifocality, pathological maximum diameter≥1, and concomitant CLNM were associated with a higher risk of LLNM.

Age is commonly incorporated into staging systems for differentiated thyroid carcinoma. Consistent with Lu et al. (16), younger PTC patients appear more prone to LLNM than older patients, possibly related to tumor biological activity and the presence of occult micrometastases. Although many staging systems include age as a prognostic indicator, the optimal age cutoff for predicting LLNM remains controversial.

Tumor size has repeatedly been associated with LLNM, with the risk generally increasing as the diameter enlarges. However, studies have reported different thresholds. Feng et al. (8)and Zhou et al. (17)suggested a cutoff >1.0 cm (18, 19); Wu et al. proposed >0.7 cm (20); and Kim et al. reported that PTC >2 cm is a strong independent predictor of LLNM (21). Ultrasound−measured tumor size reflects tumor growth. In the present study, we used 1 cm as the cutoff for both ultrasound and pathology. Further clinical studies are required to define the optimal threshold. Pathological tumor diameter has likewise been shown to predict LLNM, with reported thresholds ranging from 0.5 cm to 3.0 cm. When the diameter exceeds 1 cm—particularly ≥4 cm—the risk of lateral neck metastasis and the percentage of positive nodes increase markedly (17).

Multifocality has been recognized as a risk factor for both CLNM and LLNM in PTC, consistent with the findings by Wang et al. and with our results (22).

CLNM has also been identified as an independent predictor of LLNM (23, 24), when the number of positive central nodes exceeds three, the risk of LLNM rises substantially (25).

With respect (26, 27) to ultrasonographic nodal features, hyperechoic foci consistent with microcalcifications are highly specific for metastatic involvement, though sensitivity is relatively low (20%–30%) (28), possibly reflecting psammoma body deposition or tumor necrosis. Nodal vascularity reflects angiogenesis, and metastatic nodes often exhibit abundant or peripheral blood flow (29).

In our set, neither BRAF V600E mutation nor coexisting Hashimoto’s thyroiditis showed significant differences in univariate analysis. Some studies have identified BRAF V600E as an important biomarker of PTC progression (30), whereas Liu et al. (31)reported a higher likelihood of LLNM in BRAF−wildtype tumors.

Regarding HT, some reports suggest a protective effect against LNM (22), whereas others Wen et al. (32) identified serum TgAb as a risk factor for CLNM under the same thyroid function testing criteria, and Zhao et al. (33) found no association between antibody status and LNM. In our study, HT was determined by preoperative ultrasound and antibody levels rather than postoperative pathology, which may partly explain the lack of association.

Whether to perform prophylactic LND in cN0 patients remains controversial. Opponents argue that prophylactic LND increases operative time and postoperative complications—including recurrent laryngeal nerve injury, permanent hypoparathyroidism, chylous leakage, sympathetic chain injury, spinal accessory nerve injury, and cervical plexus neuropathic pain—without clear survival benefit (22). Proponents contend that when performed by experienced surgeons following standardized procedures, prophylactic LND reduces recurrence and reoperation rates, improves quality of life, and does not substantially increase long−term complications (14).

This study has several limitations that warrant acknowledgment. Patients with concurrent malignancies or a history of neck surgery were excluded to minimize potential confounding effects on lymph node status, while those with incomplete data were excluded to maintain the integrity of the model development process. Although these exclusions were methodologically justified, they—along with the inherent limitations of a retrospective, single-center design—may restrict the generalizability of our findings. The relatively small sample size, particularly within the validation cohort, may compromise the stability of the performance estimates and likely accounts for the observed decline in AUC values during validation. This highlights the need for cautious interpretation of the model’s predictive accuracy. Nonetheless, despite the moderate AUC in the validation set, the models demonstrate robust calibration and a favorable net benefit in decision curve analysis, indicating their potential for clinical application through reliable, individualized risk prediction. Therefore, external validation in larger, prospective, and multicenter cohorts is necessary to confirm the generalizability and reliability of the proposed nomograms. We therefore endeavored to construct accurate and objective predictive models for LLNM to aid clinicians in determining surgical strategies and delivering precision treatment. Patients with high postoperative risk scores may warrant more frequent surveillance and heightened attention to possible LLNM.

## 5 Conclusion

The decision curve analysis demonstrated a higher net clinical benefit for both models compared to the treat-all or treat-none strategies, underscoring their good predictive accuracy and clinical utility.

## Data Availability

The raw data supporting the conclusions of this article will be made available by the authors, without undue reservation.
